# Differential modulation of gentamicin antibacterial activity by vitamin B12 and vitamin D3 against Gram-positive and Gram-negative bacteria: an *in vitro–in silico* study

**DOI:** 10.1080/07853890.2026.2653307

**Published:** 2026-04-26

**Authors:** Imane M’hamedi, Ilyes Zatla, Saadia Benelhadj-Djelloul, Ibtissem Kara-Terki, Samia Bellifa, Lamia Boublenza

**Affiliations:** ^a^ Laboratory of Microbiology applied to the Food industry, Biomedical and the Environment, Faculty of Natural and Life Sciences, Earth and Universe Sciences, Department of Biology, University of Abou Bekr Belkaid, Tlemcen, Algeria; ^b^ Department of Biology, Faculty of Science and Technology, Belhaj Bouchaib University, Ain Temouchent, Algeria; ^c^ Laboratory of Bioressources Natural local, Hassiba Benbouali University, Chlef, Algeria

**Keywords:** Gentamicins, vitamin B 12, cholecalciferol, aminoglycosides, drug resistance, molecular docking

## Abstract

**Introduction:**

Antimicrobial resistance and gentamicin-associated toxicity necessitate the development of non-antibiotic adjuvants that enhance aminoglycoside efficacy while reducing required doses. Vitamins B12 and D3 possess physicochemical and biological properties that may influence bacterial resistance pathways. This study aimed to evaluate the modulatory effects of these vitamins on gentamicin against representative bacterial strains using an integrated *in vitro*–*in silico* approach.

**Materials and methods:**

Eight ATCC Gram-negative and Gram-positive reference strains were evaluated. Antibacterial activity was assessed using agar disk diffusion and broth microdilution assays to determine inhibition zones, minimum inhibitory concentrations (MICs), and MIC fold reduction (MFR). *In silico* analyses targeted the 30S ribosomal subunit, a cobalamin riboswitch, and a proton motive force (PMF)-related protein to explore mechanistic interactions underlying observed phenotypes.

**Results:**

Vitamin B12 significantly enhanced gentamicin efficacy, reducing MICs by up to 8-fold and demonstrating synergistic activity (MFR ≥ 4) against *Klebsiella pneumoniae, Pseudomonas aeruginosa, Staphylococcus aureus, and Bacillus subtilis*. Conversely, vitamin D3 displayed antagonistic effects (MFR ≤ 0.5) in several Gram-positive strains, increasing gentamicin MICs by up to 16-fold. Computational results indicated cooperative stabilization of gentamicin at the ribosomal target and increased affinity for the cobalamin riboswitch in the presence of vitamin B12, whereas vitamin D3 showed strong binding to a PMF-related protein, consistent with impaired aminoglycoside uptake.

**Conclusion:**

Vitamin B12 represents a promising dose-sparing adjuvant for gentamicin, while vitamin D3 may compromise aminoglycoside efficacy under the tested conditions. These findings warrant further validation using clinical isolates and biofilm models before considering vitamin-based strategies in aminoglycoside stewardship.

## Introduction

Antimicrobial resistance (AMR) poses a major threat to global health, driven largely by antibiotic misuse in clinical and agricultural settings [1,2]. Projections suggest AMR could cause up to 10 million deaths annually by 2050, highlighting the urgent need for strategies that preserve existing antibiotics [3,4]. One promising approach is the combination of conventional antibiotics with non-antibiotic adjuvants [5].

Aminoglycosides, particularly gentamicin, remain important antibiotics but are limited by resistance mechanisms—such as enzymatic modification and efflux—and dose-dependent toxicity, which restrict their therapeutic window [6]. These constraints underscore the need for adjuvant strategies to enhance gentamicin efficacy while mitigating toxicity.

In this context, micronutrients emerge as a promising avenue of investigation. Vitamins, particularly B12 and D3, are involved in cellular metabolism and play a fundamental role in immune regulation [7]. Beyond these systemic functions, accumulating evidence suggests that certain vitamins can directly or indirectly modulate bacterial susceptibility to antibiotics by influencing membrane properties, metabolic stress responses, or target accessibility [8].

Vitamin D3, owing to its steroidal and lipophilic nature, is known to interact with biological membranes. This property suggests a potential impact on bacterial membrane integrity and permeability [7], which is particularly relevant for aminoglycosides, whose uptake is strongly dependent on membrane potential and barrier function. Alterations in membrane dynamics may therefore influence gentamicin internalization and activity.

In parallel, vitamin B12 is an essential cofactor in prokaryotic metabolic pathways, including those involved in energy production and redox balance. Perturbation of B12 homeostasis has been shown to sensitize bacteria to antibiotic-induced stress [9]. Given that aminoglycoside efficacy is closely linked to bacterial metabolic state, B12-mediated metabolic modulation represents a plausible mechanism for altering gentamicin activity.

Several studies have already demonstrated notable antimicrobial activity for other vitamins (K, C, E), both alone and in combination with antibiotics [10–12]. In contrast, while intrinsic antimicrobial activity has been reported for vitamin [13], comparable evidence for a direct antibacterial effect of vitamin B12 is scarce. Critically, the capacity of either vitamin to modulate the activity of gentamicin has, to our knowledge, never been investigated, leaving the nature of this interaction (whether synergistic, antagonistic, or additive) an open scientific question. To advance beyond simple phenotypic observation and probe potential mechanisms, an *in silico* approach is particularly relevant, as molecular docking allows evaluation of the structural plausibility of direct interactions between these vitamins and bacterial protein targets, providing a predictive framework for interpreting experimental results.

Therefore, the objective of this study is to test the central hypothesis that vitamin B12 enhances, whereas vitamin D3 attenuates, the antibacterial efficacy of gentamicin through differential modulatory interactions at bacterial molecular targets. To this end, we provide the first systematic characterization of the interactions between vitamins D3, B12, and gentamicin using a combined *in vitro*–*in silico* approach. Specifically, we aim (i) to quantitatively determine the modulatory effects of vitamin D3–gentamicin and vitamin B12–gentamicin combinations on sensitive reference strains, and (ii) to explore, *via* molecular docking, the structural underpinnings of observed interactions by predicting binding affinities for key bacterial targets, thereby generating mechanistic hypotheses to inform future studies on resistant isolates and dose-reduction strategies.

## Methods

### Source of vitamins and antimicrobial agent

The test agents were cholecalciferol (vitamin D3), cyanocobalamin (vitamin B12), and gentamicin. Vitamin D3 was used as Vitamine D3 B.O.N^®^ (oily solution, 200,000 IU/mL, corresponding to 5000 µg/mL; Bouchara-Recordati, France). Vitamin B12 was used as Vitamin B12 RAZES^®^ (aqueous solution, 500 µg/mL; Frater-Razes, Algeria). Gentamicin was used in two forms: 30 µg diffusion discs (Bio-Rad^®^, France) for agar assays, and a 10 mg/mL stock solution for microdilution tests, prepared by dissolving pure gentamicin sulfate powder (Sigma-Aldrich, USA) in sterile distilled water and sterilizing the solution through a 0.22 µm filter.

Vitamin B12 and vitamin D3 concentrations were selected based on reported physiological and pharmacological ranges, as well as prior *in vitro* studies examining vitamin–microbe or vitamin–drug interactions [14–16]. Although systemic serum concentrations of these vitamins are tightly regulated *in vivo*, higher localized or transient tissue-level concentrations may occur, particularly under supplementation or therapeutic conditions [14,16]. Importantly, the concentrations used here were not intended to directly mimic plasma levels but rather to probe concentration-dependent modulatory trends and generate mechanistic hypotheses that can inform future translational and pharmacokinetic investigations [15,16].

### Bacterial strains and culture conditions

Eight reference strains were obtained from the American Type Culture Collection (ATCC). The panel included three Gram-negative species (*Escherichia coli* ATCC 25922*, Pseudomonas aeruginosa* ATCC 27853*, Klebsiella pneumoniae* ATCC 700603) and five Gram-positive species (*Staphylococcus aureus* ATCC 6538*, Micrococcus luteus* ATCC 15307*, Enterococcus faecalis* ATCC 49452*, Bacillus subtilis* ATCC 6633*, and Bacillus cereus* ATCC 10876).

The eight ATCC reference strains included in this study were selected to provide a standardized and reproducible panel encompassing both Gram-positive and Gram-negative bacteria with well-characterized susceptibility profiles. These strains are widely used as benchmark organisms in antimicrobial and pharmacodynamic studies, enabling direct comparison with existing literature and ensuring experimental reproducibility.

### *In vitro* antibacterial susceptibility testing

#### Disk diffusion susceptibility screening

Antibacterial activity was first assessed using the agar diffusion method [17]. A bacterial suspension was prepared from a fresh overnight culture and adjusted to a 0.5 McFarland turbidity standard (10^8^ CFU/mL). This suspension was spread onto Mueller–Hinton agar plates to obtain a uniform lawn. Sterile 6 mm filter paper discs (Whatman No. 1) were impregnated with 20 µL of either the vitamin B12 solution (500 µg/mL) or the vitamin D3 solution (5000 µg/mL). For combination testing, commercial 30 µg gentamicin discs were supplemented with 20 µL of the vitamin B12 solution or 10 µL of the vitamin D3 solution, whereas unmodified 30 µg gentamicin discs served as positive controls. After incubation for 18–24 h at 37 °C, inhibition zone diameters were measured in millimeters. Differences between combination discs and gentamicin-only controls were analyzed using a Tukey’s test, with a p-value < 0.05 considered significant.

#### Determination of MICs and assessment of modulatory effect

Antibacterial activity was quantified by the broth microdilution method, following CA-SFM/EUCAST recommendations [18]. The final inoculum in each well was adjusted to 5 × 10^5^ CFU/mL by diluting a 0.5 McFarland suspension prepared from a fresh overnight culture. In 96-well plates, the MIC of gentamicin (serial twofold dilutions from 128 to 0.0625 µg/mL) was determined in parallel either alone or in the presence of a fixed, experimentally confirmed non-inhibitory concentration of vitamin D3 (1250 µg/mL) or vitamin B12 (125 µg/mL). To maintain vitamin D3 solubility, 0.1% (v/v) Tween 80 was added to all relevant test and control wells. Plates were incubated for 18 h at 37 °C, and MICs were defined as the lowest concentration preventing visible growth. All MIC determinations were performed in triplicate.

The modulatory effect was expressed as the MIC fold reduction (MFR), calculated as:

$$\text{MFR}=\left(\text{MIC of gentamicin alone}\right)/\left(\text{MIC of gentamicin in the presence of the vitamin}\right).$$

Interactions were classified according to established criteria for synergy screening [18,19]: synergy, MFR ≥ 4; additive effect or indifference, 0.5 < MFR < 4; and antagonism, MFR ≤ 0.5.

Synergy was defined as *a* ≥ 4-fold reduction in MIC (MFR ≥ 4) for the combination compared with the most active single agent, consistent with commonly used checkerboard/FIC index interpretation frameworks and international susceptibility testing recommendations [20,21].

### *In silico* study

#### Molecular docking

Molecular docking was performed to investigate the binding of vitamin B12, vitamin D3, and gentamicin to bacterial targets implicated in transport, resistance, and antibiotic action. Three primary targets were selected for the same bacteria studied *in vitro*: the 30S ribosomal subunit (16S rRNA, PDB ID: 7OE1), the cobalamin riboswitch (PDB ID: 6VMY), and a proton-motive-force (PMF)-related protein (PDB ID: 6NWD), involved in maintaining the proton gradient across the membrane and is critical for energy production and transport processes in bacterial cells [22]. Protein structures were retrieved from the Protein Data Bank and prepared in AutoDock Tools 1.5.7 by removing crystallographic water molecules, adding polar hydrogens, and assigning Gasteiger charges. Ligands (gentamicin, vitamin B12, and vitamin D3) were downloaded from PubChem and energy-minimized using the MMFF94 force field in Avogadro. Docking was carried out with AutoDock Vina 1.2.5 using grid boxes centered on known binding sites: the A-site/decoding center of the 30S subunit, the ligand-binding pocket of the B12 aptamer domain, and the transmembrane cavity of the PMF-related protein. Each ligand was docked individually, and for the ribosome, gentamicin and vitamin B12 were also docked simultaneously to explore potential competitive versus cooperative binding. For each run, ten poses were generated and ranked by binding affinity, and interactions were analyzed in Biovia Discovery Studio.

#### Molecular dynamics simulations

To further assess the stability and potential cooperativity of gentamicin and vitamin B12 binding to the 30S ribosomal subunit, molecular dynamics simulations of 100 ns were performed using GROMACS 2023 with the CHARMM36 force field for the protein and CGenFF-derived topologies for the ligands [23]. Protein–ligand complexes were placed in a triclinic box with at least 1.0 nm between solute and box edge and solvated with the TIP3P water model. Sodium and chloride ions were added to neutralize the system and reach a physiological ionic strength of 0.15 M. Energy minimization was performed using the steepest-descent algorithm until the maximum force was below 1000 kJ/mol/nm. Equilibration consisted of a 100 ps NVT phase at 310 K using the V-rescale thermostat, followed by a 100 ps NPT phase at 1 bar using the Parrinello–Rahman barostat. Production runs were carried out for 100 ns with a 2 fs time step. Long-range electrostatics were treated with the Particle Mesh Ewald method, and all covalent bonds were constrained using the LINCS algorithm. Trajectories were analyzed to calculate root-mean-square deviation (RMSD), root-mean-square fluctuation (RMSF), radius of gyration (Rg), and hydrogen-bonding profiles.

## Results

### Qualitative assessment of antibacterial interactions by agar disk diffusion

The antibacterial activities of gentamicin, vitamin B12, and vitamin D3 were first evaluated individually and in combination using the agar disk diffusion method. When tested alone, neither vitamin B12 nor vitamin D3 produced inhibition zones against any of the eight reference strains, indicating an absence of intrinsic antibacterial activity under the tested conditions. In contrast, gentamicin alone was active against seven of the eight strains, with *E. faecalis* (ATCC 49452) showing the lowest susceptibility (17.3 ± 0.57 mm).

The modulatory effects of the vitamins on gentamicin activity are summarized in Table 1. The combination of gentamicin with vitamin B12 produced a statistically significant increase in inhibition zone diameter for five strains. The largest increases were observed for *P. aeruginosa* (+6.0 mm), *S. aureus* (+4.3 mm), and *B. subtilis* (+4.3 mm) (*p* < 0.05 for all). For the remaining three strains (*M. luteus, E. faecalis*, and *B. cereus*), the addition of vitamin B12 did not significantly modify the inhibition zone diameter (*p* > 0.05).

**Table 1.** Inhibition zone diameters of gentamicin alone and in combination with vitamins against reference bacterial strains.

Bacterial strain	Zone diameter (mm) Gen alone	Zone diameter (mm) Gen + Vit B12	*p* Value	Zone diameter mm Gen + Vit D3	*p* Value
*E.coli* (ATCC25922)	20.3 ± 0.57	24 ± 1.00	0.015*	19.6 ± 1.52	0.751
*K. pneumoniae* (ATCC 700603)	19.3 ± 0.57	23.3 ± 1.52	0.021*	20.3 ± 1.52	0.632
*P. aeruginosa* (ATCC 27853)	20.6 ± 0.57	26.6 ± 1.52	0.012*	20.6 ± 2.51	1
*S. aureus* (ATCC 6538)	23.3 ± 0.57	27.6 ± 1.52	0.007*	20 ± 1.00	0.023*
*M. luteus* (ATCC 15307)	27 ± 1.00	28 ± 1.00	0.726	18.6 ± 1.52	0.001*
*E. faecalis* (ATCC 49452)	17.3 ± 0.57	18.3 ± 1.52	0.569	12.3 ± 1.15	0.004*
*B. subtilis* (ATCC 6633)	25 ± 1.00	29.3 ± 2.08	0.019*	23.3 ± 2.08	0.828
*B. cereus* (ATCC 10876)	26.6 ± 0.57	28.6 ± 2.08	0.455	21.3 ± 2.51	0.033*

Data are presented as the mean ± standard deviation (SD) of three independent experiments. An asterisk (*) indicates a statistically significant difference (*p* < 0.05) compared to the gentamicin-only control, as determined by Tukey’s test.

In contrast, combining gentamicin with vitamin D3 led to a statistically significant reduction in inhibition zone diameter for four of the eight strains. The greatest decreases were recorded for *M. luteus* (−8.4 mm), *B. cereus* (−5.3 mm), and *E. faecalis* (−5.0 mm) (*p* < 0.05 for all). For the remaining four strains, including all three Gram-negative species, vitamin D3 did not significantly affect gentamicin activity (*p* > 0.05).

### Quantitative assessment of modulatory effects on gentamicin MICs

To quantify these interactions, the MICs of gentamicin were determined alone and in the presence of a fixed, non-inhibitory concentration of vitamin B12 or vitamin D3, and the MIC fold reduction (MFR) was calculated (Table 2). Addition of vitamin B12 reduced the gentamicin MIC for five of the eight strains. A synergistic interaction (MFR ≥ 4) was observed for four strains: *K. pneumoniae* (8-fold reduction, from 4 to 0.5 µg/mL; MFR = 8), *P. aeruginosa* (4-fold reduction, from 0.5 to 0.125 µg/mL; MFR = 4), and both *S. aureus* and *B. subtilis* (4-fold MIC reductions). For *E. coli*, the MIC decreased 2-fold (MFR = 2), indicating a modest effect below the synergy threshold, while *M. luteus, E. faecalis*, and *B. cereus* showed no MIC change (MFR = 1), consistent with indifference.

**Table 2.** Modulatory effect of vitamin B12 and vitamin D3 on the minimum inhibitory concentration (MIC) of gentamicin against reference bacterial strains.

Bacterial strain	MIC (µg/mL) Gen alone	MIC (µg/mL) Gen + Vit B12	MFR	MIC (µg/mL) Gen + Vit D3	MFR
*E.coli* (ATCC25922)	4	2	2	4	1
*K. pneumoniae* (ATCC 700603)	4	0.5	8	4	1
*P. aeruginosa* (ATCC 27853)	0.5	0.125	4	0.5	1
*S. aureus* (ATCC 6538)	2	0.5	4	16	0.125
*M. luteus* (ATCC 15307)	1	1	1	8	0.125
*E. faecalis* (ATCC 49452)	16	16	1	128	0.125
*B. subtilis* (ATCC 6633)	2	0.5	4	4	0.5
*B. cereus* (ATCC 10876)	0.0625	0.0625	1	1	0.0625

Combination assays were performed with a fixed concentration of Vitamin B12 (125 µg/mL) or Vitamin D3 (1250 µg/mL). The MIC Fold Reduction (MFR) was calculated as (MIC of gentamicin alone)/(MIC of gentamicin in combination). MFR values indicating synergy (≥4), indifference, 0.5 < MFR < 4 or antagonism (≤0.5).

In the presence of vitamin D3, gentamicin activity was not enhanced for any strain. Instead, significant antagonism (MFR ≤ 0.5) was observed for five strains. The strongest antagonism occurred for *S. aureus, M. luteu*s, and *E. faecalis*, each exhibiting an 8-fold MIC increase (MFR = 0.125). *B. cereus* showed a 16-fold increase in MIC (from 0.0625 to 1 µg/mL; MFR = 0.0625), while *B. subtilis* displayed a 2-fold increase (MFR = 0.5), meeting the threshold for antagonism. For the three Gram-negative strains (*E. coli, K. pneumoniae*, and *P. aeruginosa*), the MICs of gentamicin remained unchanged in the presence of vitamin D3 (MFR = 1).

### Molecular docking

Molecular docking was performed to characterize the interactions of vitamin B12, gentamicin, and vitamin D3 with key bacterial targets. The 30S ribosomal subunit (16S rRNA, PDB ID: 7OE1) and the cobalamin riboswitch (PDB ID: 6VMY) were selected as primary binding sites for vitamin B12 and gentamicin, while a proton-motive-force (PMF)–related protein (PDB ID: 6NWD) was chosen as the target for vitamin D3. Binding affinities and residue-level contacts are summarized in Table 3.

**Table 3.** Molecular docking results of vitamin B12 and gentamicin against the 30S ribosomal subunit (16S rRNA, PDB ID: 7OE1) and the cobalamin riboswitch (PDB ID: 6VMY).

Target	Ligand	Docking Score (kcal/mol)	Interacting residues	Types of interaction
30S ribosomal subunit (16S rRNA, PDB ID: 7OE1)	Vitamin B12	−9.54	Gln8 Ile11 Gly18 Glu59 Val93 Glu20 Lys24	2 H-bonds H-bond H-bond H-bond H-bond 2 H-bonds + Ionic bond H-bond + Ionic bond
	Gentamicin	−6.64	Arg52 Arg110 Asn121 Asp112 Glu122 Glu128 Asp125	H-bond H-bond H-bond Ionic bond Ionic bond Ionic bond 2 H-bonds + Salt bridge
Cobalamin riboswitch (PDB ID: 6VMY)	Vitamin B12	−8.74	G222 A231 A303 A306	H-bond H-bond H-bond Ionic bond
	Gentamicin	−9.31	G246 A231 G232 C233 C234 A243 G244 G245	H-bond H-bond + Ionic bond Salt bridge Ionic bond Ionic bond Ionic bond H-bond + Salt bridge H-bond + Salt bridge

Docking scores are expressed in kcal/mol, interacting residues and the types of interactions are listed for each ligand–target complex.

**Figure 1.**
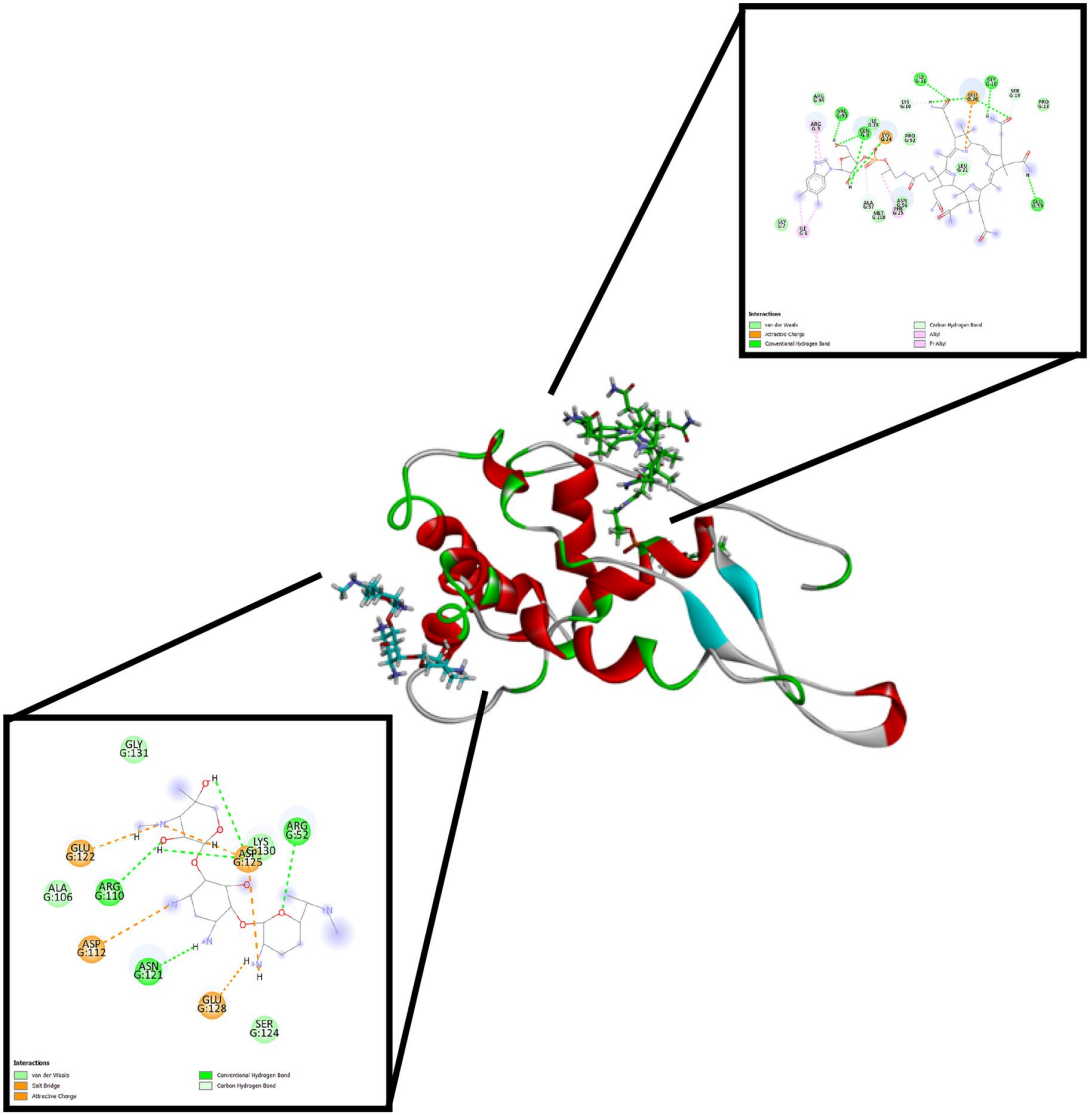
Docking interactions of Vitamin B12 (Green sticks) and Gentamicin *Cyan sticks) with the 30S ribosomal subunit (16S rRNA, PDB ID: 7OE1).

For the 30S ribosomal subunit, vitamin B12 showed a higher binding affinity (−9.54 kcal/mol) than gentamicin (−6.64 kcal/mol). Vitamin B12 formed an extensive network of hydrogen bonds and ionic interactions with Gln8, Ile11, Gly18, Glu20, Glu59, Val93, and Lys24, indicating a strongly anchored pose in the ribosomal binding pocket. Gentamicin also bound to the ribosome but through fewer stabilizing interactions, relying mainly on hydrogen bonds with Arg52, Arg110, and Asn121, and ionic contacts with Asp112, Glu122, Glu128, and Asp125. Both ligands were located at or near the decoding center of the 30S subunit, consistent with the known aminoglycoside binding region. Figure 1 illustrates that vitamin B12 and gentamicin occupy distinct but neighboring sites within this area, each forming specific, stable interactions.

**Figure 2.**
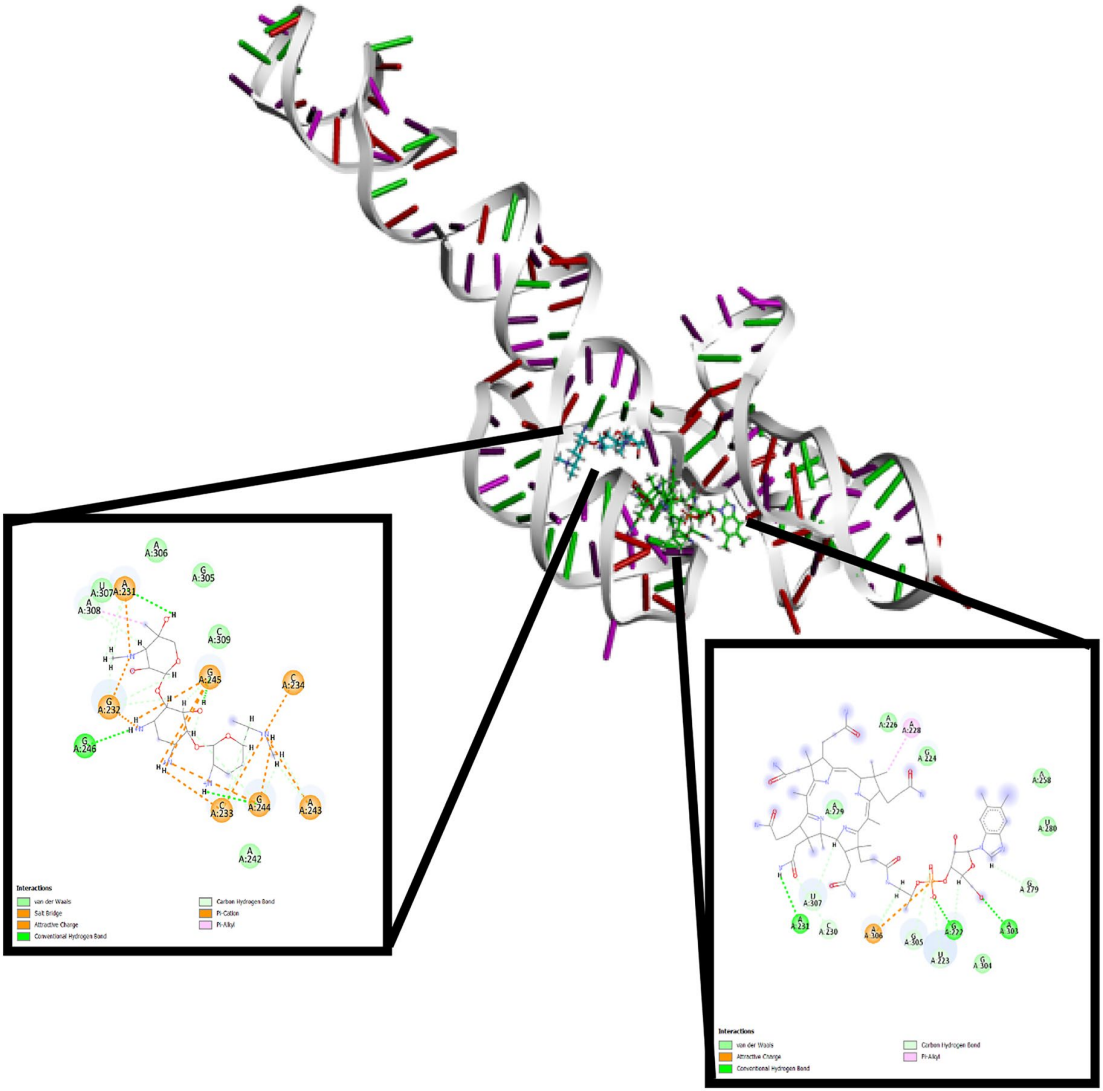
Docking interactions of Vitamin B12 (Green sticks) and Gentamicin (blue sticks) with the cobalamin riboswitch (PDB ID: 6VMY).

In the cobalamin riboswitch domain, vitamin B12 exhibited a binding affinity of −8.74 kcal/mol and interacted with G222, A231, A303, and A306 *via* hydrogen bonds and an ionic interaction. Gentamicin displayed an even stronger binding score (−9.31 kcal/mol), contacting G246, A231, G232, C233, C234, A243, G244, and G245 through hydrogen bonds, ionic bonds, and salt bridges (Figure 2, Table 3).

**Figure 3.**
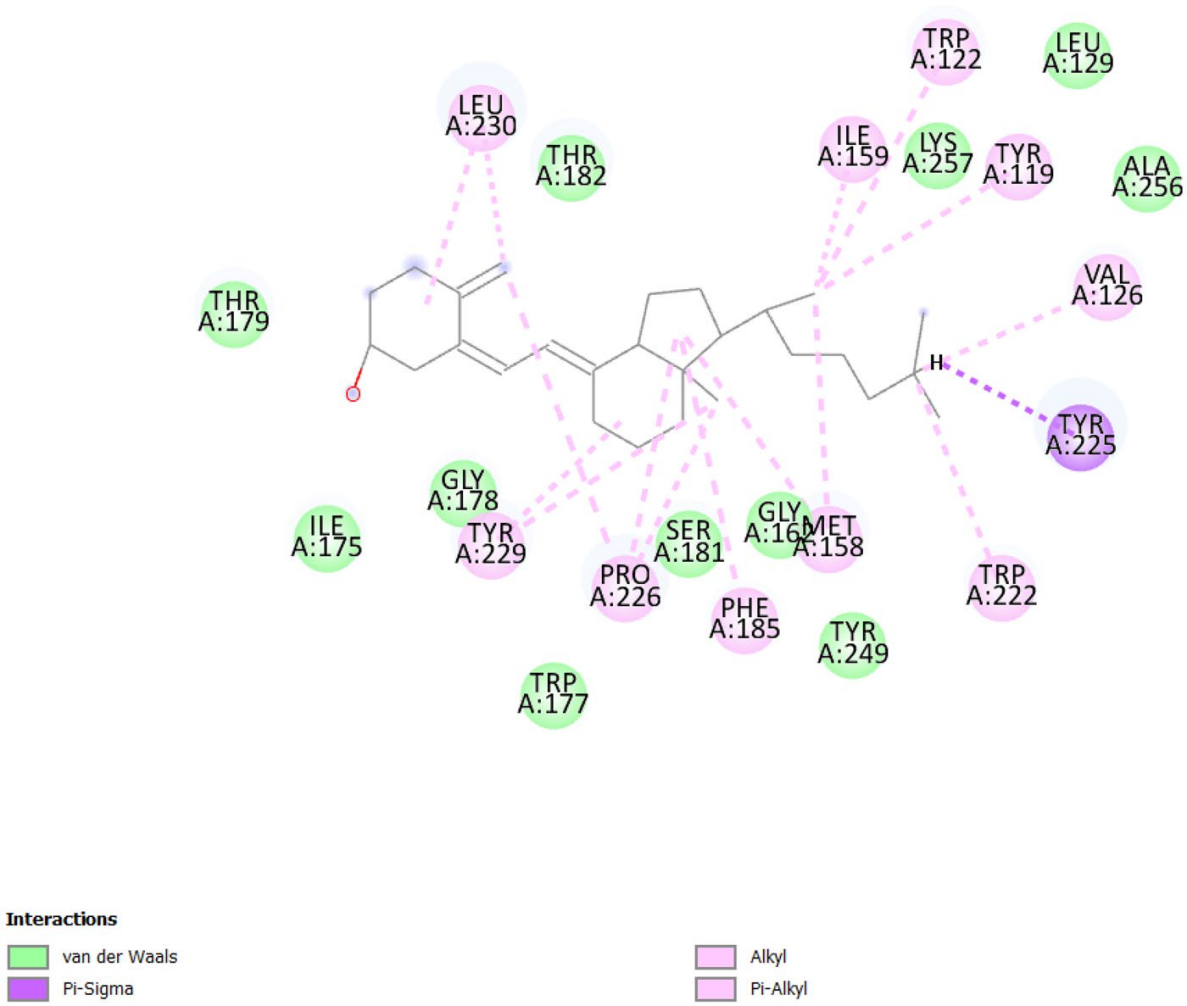
2D interaction of Vitamin D within the binding pocket of the PMF-related protein (PDB ID: 6NWD). The ligand forms predominantly hydrophobic and van der Waals interactions, with a characteristic π–σ interaction involving Tyr225, contributing to the strong binding affinity (–10.10 kcal/mol).

Vitamin D3 showed the strongest binding affinity among all tested ligands toward the PMF-related protein, with a docking score of −10.10 kcal/mol. The vitamin D3–PMF complex was stabilized predominantly by hydrophobic and van der Waals interactions, including a characteristic π–σ contact with Tyr225 (Figure 3). The interaction pattern was largely non-polar, suggesting deep insertion of vitamin D3 into the hydrophobic binding pocket of the protein.

### Molecular dynamics

**Figure 4.**
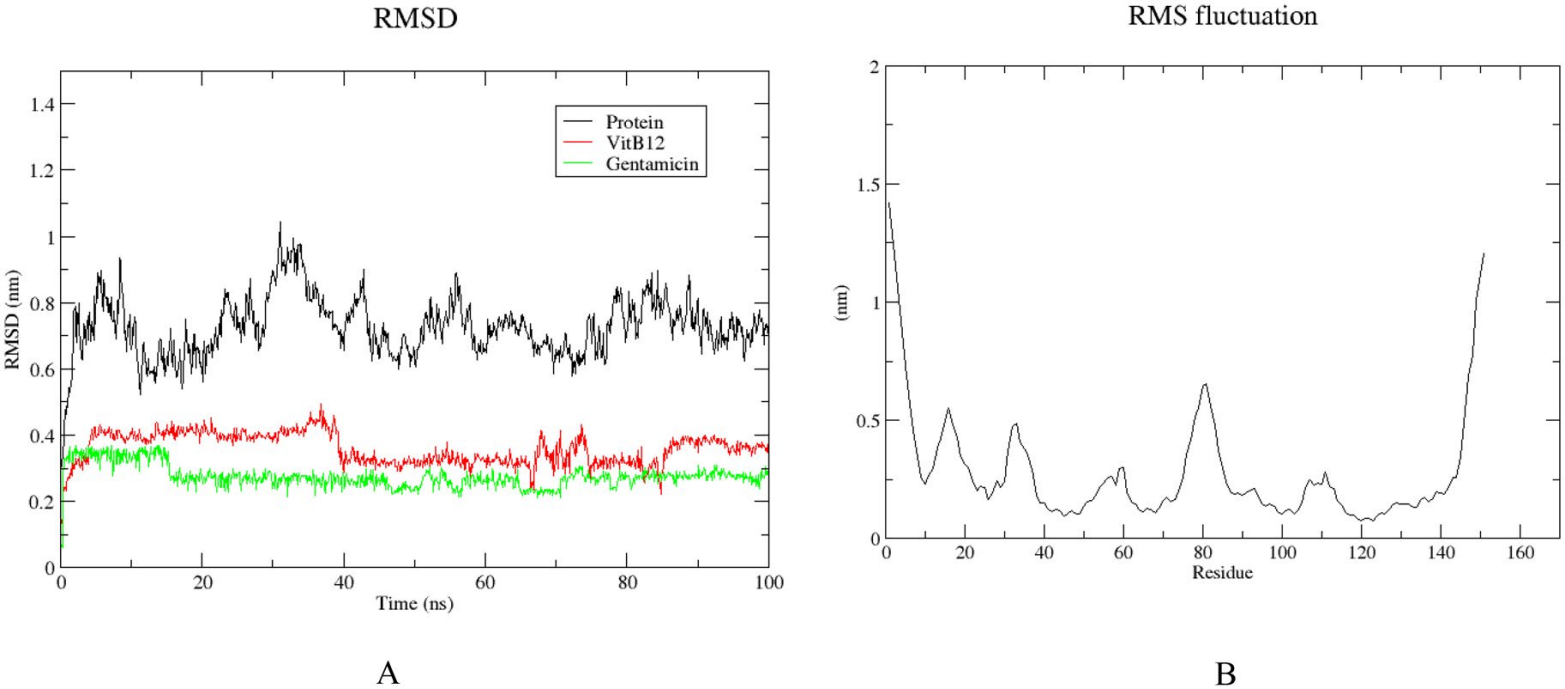
Molecular dynamics analysis of the 30S ribosomal subunit (16S rRNA, PDB ID: 7OE1) bound to Vitamin B12 and Gentamicin. (A) RMSD plots for the ribosomal protein backbone (black), Vitamin B12 (red), and Gentamicin (green), showing overall stability throughout the 100 ns simulation. (B) RMSF profile of ribosomal residues.

**Figure 5.**
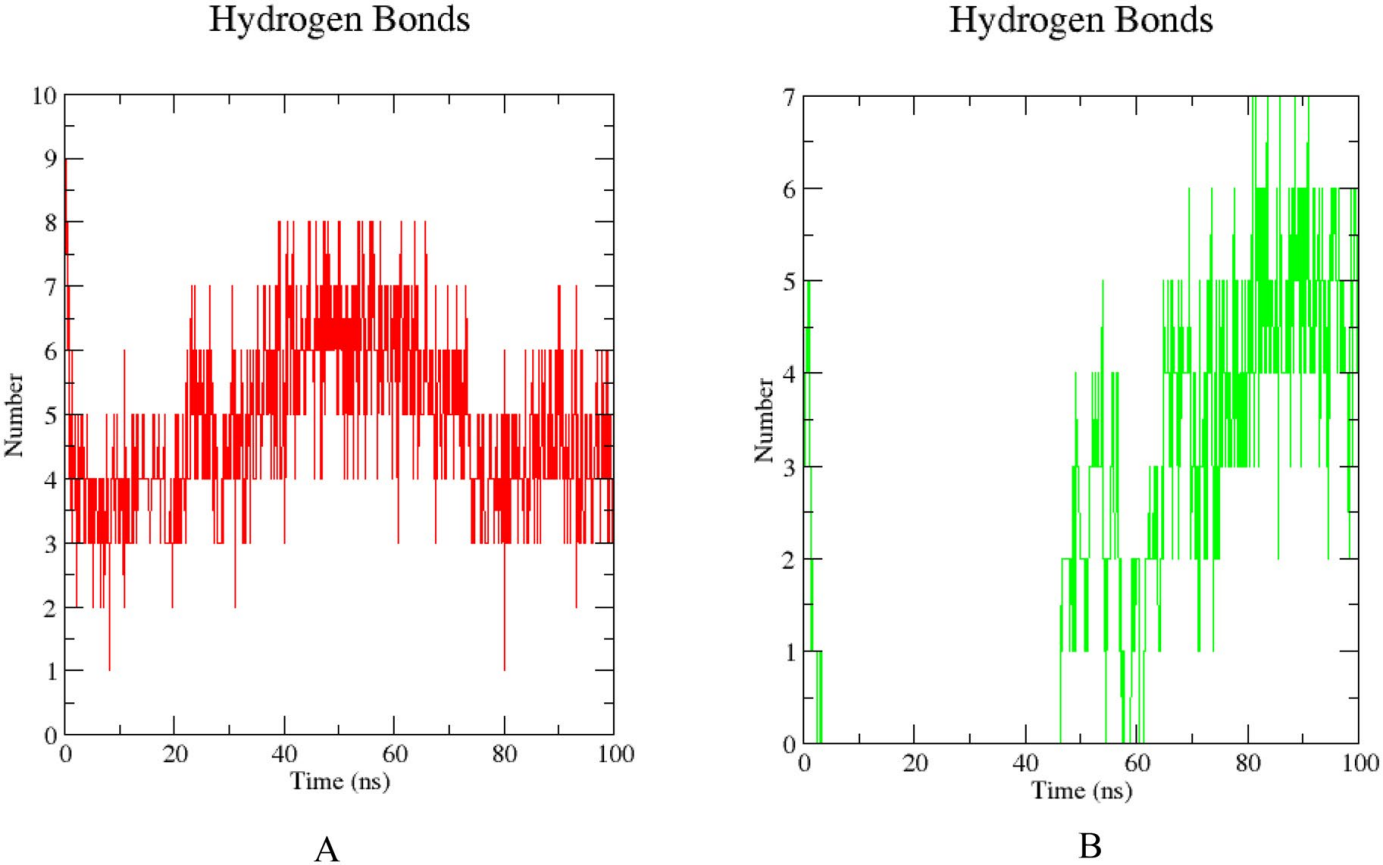
Hydrogen bond dynamics between ligands and the ribosomal subunit during the 100 ns simulation. (A) between Vitamin B12 and ribosomal residues (B) between Gentamicin and ribosomal residues.

Molecular dynamics results are presented to evaluate the dynamic stability of the ribosomal complexes and the feasibility of co-binding, 100 ns molecular dynamics simulations were carried out for the 30S ribosomal subunit (PDB ID: 7OE1) in complex with vitamin B12 and gentamicin. System stability and local flexibility were assessed using root-mean-square deviation (RMSD), root-mean-square fluctuation (RMSF), and hydrogen-bond analyses [24,25] (Figures 4 and 5).

The protein backbone RMSD remained between 0.6 and 1.0 nm throughout the 100 ns trajectory, indicating overall conformational stability of the ribosomal framework. Vitamin B12 showed a highly stable trajectory, with RMSD values centered around 0.40 ± 0.05 nm, reflecting strong anchoring in its binding pocket. Gentamicin also remained stably bound, with RMSD values of approximately 0.30 ± 0.01 nm (Figure 4(A)).

RMSF analysis revealed relatively low flexibility in residues surrounding the decoding center. In the presence of both vitamin B12 and gentamicin, RMSF values in this region were reduced compared with gentamicin alone, consistent with local stabilization of the binding environment (Figure 4(B)). Throughout the simulation, vitamin B12 maintained a network of 4–8 hydrogen bonds with ribosomal residues (Figure 5(A)), whereas gentamicin maintained 5–6 hydrogen bonds toward the end of the trajectory (Figure 5(B)). The persistence of hydrogen bonding for both ligands indicates that simultaneous binding is structurally compatible and that both complexes remain stable over the simulation timescale.

## Discussion

The pursuit of adjuvants to overcome antimicrobial resistance is a global priority, but its success depends on a detailed mechanistic understanding of drug interactions [26,27]. This study dissected the interplay between two key vitamins, B12 and D3, and gentamicin using an integrated *in vitro* and *in silico* approach. A first foundational finding is the absence of intrinsic antibacterial activity from either vitamin against the reference strains, as shown by the lack of inhibition zones in agar diffusion assays. This baseline is critical, because it indicates that the pronounced shifts in gentamicin efficacy arise from genuine interactions with its antibacterial pathway, primarily affecting antibiotic accumulation or target engagement.

Our observations for vitamin B12 are consistent with the broader scientific consensus that it functions as an essential metabolic cofactor and signaling molecule rather than as a classical antimicrobial agent [28]. Precisely because B12 is biologically “innocent” in a bactericidal sense, the strong synergy observed (up to an 8-fold reduction in gentamicin MIC for *Klebsiella pneumoniae* and 4-fold reductions for *Pseudomonas aeruginosa*, *Staphylococcus aureus*, and *Bacillus subtilis*) must be mediated by indirect mechanisms enhancing gentamicin activity. This aligns with reports of variable but often positive modulatory effects of B-group vitamins on antibiotic efficacy [29–32]. Our docking and molecular dynamics data provide a coherent mechanistic model: vitamin B12 binds to the 30S ribosomal subunit with higher affinity than gentamicin and stabilizes the decoding center, as evidenced by lower RMSD values and reduced RMSF of key residues in the presence of B12. This supports a cooperative binding scenario in which B12’s stable anchoring (binding affinity −9.54 kcal/mol) pre-organizes the ribosomal environment for aminoglycoside binding, consistent with structural work showing that decoding-site stabilization enhances aminoglycoside action [33].

A second, and perhaps more novel, level of modulation emerges from the cobalamin riboswitch. Docking revealed that gentamicin binds this RNA element with even higher affinity than vitamin B12 (−9.31 vs. −8.74 kcal/mol), engaging multiple nucleotides through hydrogen bonds and salt bridges. This suggests that gentamicin could act as a “decoy” ligand, altering riboswitch-mediated control of btu transport genes and potentially driving overexpression of B12 transporters. Such a mechanism is supported by studies showing that non-cognate ligands can hijack riboswitches and rewire gene expression programs involved in transport and resistance [34,35]. Together, these structural insights provide a plausible explanation for why B12–gentamicin combinations produce strong MIC reductions without any intrinsic bactericidal activity of B12 itself.

In contrast, vitamin D3 presents a more nuanced and “Janus-faced” profile. Although its potential intrinsic antimicrobial capacity is still under investigation [15], most direct effects have been reported at relatively high MICs or in specific organisms, such as membrane disruption in *Candida albicans* [13] and inhibition of oral *Streptococcus* species at 60–250 µg/mL [36]. In our conditions, no intrinsic activity was observed even at the highest concentrations tested, underscoring the importance of strain background, formulation, and methodology in shaping biological outcomes. Within this experimentally defined context, the antagonism we observed must therefore reflect interference with gentamicin’s antibacterial pathway rather than direct bacterial killing by vitamin D3.

Our *in vitro* data show that vitamin D3 significantly increases gentamicin MICs (up to 16-fold for *Bacillus cereus*) and reduces inhibition zones for several Gram-positive species, indicating a robust antagonistic effect. This finding mirrors the antagonism reported by Masadeh et al. [37] between vitamin D and ciprofloxacin and contrasts with studies where vitamin D3 synergized with streptomycin against *P. aeruginosa* biofilms or restored susceptibility in resistant isolates [38,39]. Docking provides a unifying explanation for this apparent discrepancy: vitamin D3 exhibits high-affinity binding (−10.10 kcal/mol) to a PMF-related protein *via* predominantly hydrophobic interactions, including a characteristic π–σ contact with Tyr225. Because aminoglycoside uptake is a PMF-dependent active process, D3 binding to this target is likely to impair proton-motive-force–driven transport, reducing intracellular gentamicin accumulation. This mechanistic interpretation is congruent with classical work showing that disruption of membrane potential or PMF abolishes aminoglycoside uptake and activity [40].

Juxtaposed with literature describing both synergy and antagonism for vitamin D3, our results support a context-dependent model: vitamin D3 may antagonize aminoglycosides in planktonic, susceptible cells by blocking PMF-dependent entry, while under biofilm or resistant conditions its membrane and signaling effects could instead favor antibiotic action [38,41]. This dual behavior fits the broader picture of prevalent synergy and antagonism among antimicrobials and between antimicrobials and non-antibiotic drugs [18,42,43]. A distinctive contribution of our work is to reveal this duality within a single micronutrient class: vitamin B12 and vitamin D3, both widely used supplements, drive gentamicin responses in opposite directions.

The therapeutic implications are considerable, especially in view of gentamicin’s well-recognized nephrotoxicity. The synergy observed with vitamin B12 is aligned with the goal of developing “dose-sparing” regimens that maintain efficacy while reducing aminoglycoside exposure [43,44]. This is particularly relevant given animal data showing that cobalamin can mitigate gentamicin-induced kidney injury through antioxidant and anti-inflammatory mechanisms [45,47]. Conversely, vitamin D3′s antagonism represents a latent clinical risk: reduced antibacterial response could prompt clinicians to escalate gentamicin doses, thereby increasing nephrotoxicity, as suggested by studies on complex interactions between vitamin D analogues, gentamicin, and renal function [48,49]. Collectively, our findings reframe these common vitamins as pharmacologically active agents capable of shifting the therapeutic index of gentamicin rather than as inert nutritional supplements.

Several limitations must be acknowledged. First, we used pharmaceutical formulations of the vitamins; although this reflects real-world usage and has precedent in the literature [13], confirmation with pure compounds is essential. Also, only ATCC reference strains were used, which may not fully represent the genetic diversity and multidrug-resistant profiles of clinical isolates. Second, our *in silico* mechanisms remain hypotheses that require targeted validation. Reporter assays for btuB expression would directly test the proposed riboswitch “decoy” effect, while PMF measurements using membrane-potential-sensitive dyes and gentamicin uptake assays are needed to confirm the predicted transport inhibition by vitamin D3. Third, our experiments were performed on planktonic reference strains; large-scale screens and clinical studies have shown that drug–drug interaction profiles can change markedly in clinical isolates, biofilms, and different environmental conditions [35,42]. Finally, host-level effects of vitamins, particularly the immunomodulatory roles of vitamin D3, add an additional layer of complexity that cannot be captured *in vitro* [50].

In summary, this integrated *in vitro* and *in silico* study reveals the mechanistic basis of a striking duality: vitamin B12 consistently potentiates gentamicin, whereas vitamin D3 frequently antagonizes it, through distinct target interactions at the ribosome, riboswitch, and PMF-related proteins. These results highlight the need to evaluate putative adjuvants with strict contextual rigor and caution against assuming that widely used vitamins are pharmacologically neutral during aminoglycoside therapy. Future work should extend these findings to resistant clinical isolates and biofilm models to better assess aminoglycoside efficacy under clinically relevant conditions, and to *in vivo* infection systems, to determine whether B12-based potentiation can be safely exploited and whether D3-associated antagonism warrants active monitoring or avoidance in patients receiving gentamicin.

## Conclusion

This study shows that vitamin B12 and D3 modulate gentamicin activity in opposite ways. Vitamin B12 acts as a cooperative adjuvant, particularly against Gram-negative bacteria, consistent with *in vitro* synergy and engagement of the cobalamin riboswitch. In contrast, vitamin D3 acts as an antagonist, likely impairing gentamicin uptake *via* PMF-related proteins. These findings are context-dependent and highlight the need for cautious evaluation of vitamin D3. Limitations include the use of reference strains and fixed vitamin concentrations, and future studies should examine clinical isolates, biofilm models, and dose-dependent effects.

## Data Availability

All data generated or analyzed during this study are included in this published article.
